# Ten quick tips to get you started with Bayesian statistics

**DOI:** 10.1371/journal.pcbi.1012898

**Published:** 2025-04-10

**Authors:** Olivier Gimenez, Andy Royle, Marc Kéry, Chloé R. Nater

**Affiliations:** 1 CEFE, Univ Montpellier, CNRS, EPHE, IRD, Montpellier, France; 2 U.S. Geological Survey, Eastern Ecological Science Center, Laurel, Maryland, United States of America; 3 Swiss Ornithological Institute, Sempach, Switzerland; 4 Norwegian Institute for Nature Research, Trondheim, Norway; SIB Swiss Institute of Bioinformatics, SWITZERLAND

## Introduction

Bayesian statistics is a framework in which our knowledge about unknown quantities of interest (especially parameters) is updated with the information in observed data, though it can also be viewed as simply another method to fit a statistical model. It has become popular in many branches of biology [1,2]. For context, 5 of the 10 most cited papers in Web of Science with keywords ‘Bayesian statistics’ are related to biology (as of August 19, 2024). The use of Bayesian statistics in biology allows researchers to run analyses that incorporate external knowledge, describe complex systems, and work effectively with limited or messy data. However, most biologists are first trained in frequentist statistics. Learning to become fluent in Bayesian statistics may be perceived as too time-consuming to undertake, or the prospect of adopting an unfamiliar statistical framework can simply appear too daunting. Despite this perception, however, the learning curve for Bayesian statistics is gradual, not steep, and benefits will quickly outweigh investments.

**Fig 1:**
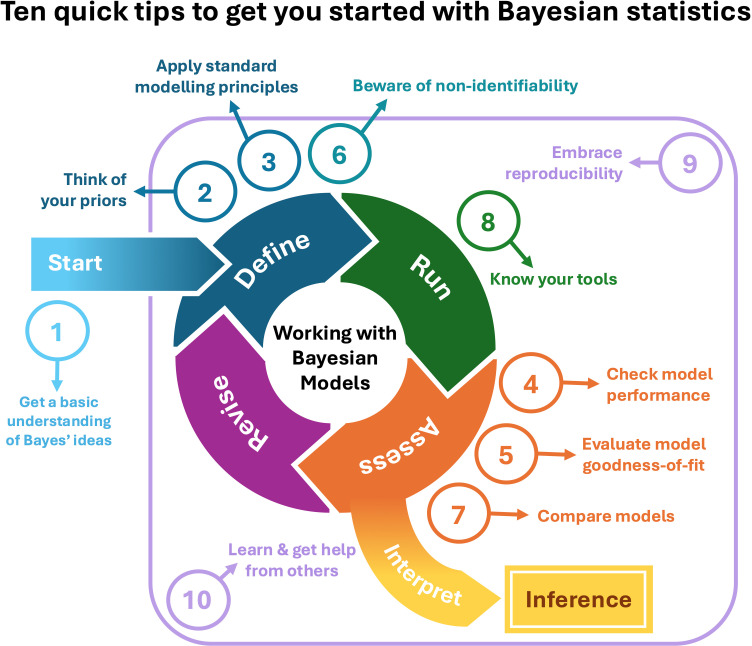
Graphical summary of 10 quick tips to get started with Bayesian statistics and how they fit into a larger view of an analytical workflow with Bayesian models. Because some tips pertain to overarching concepts and best practices, and others are more specific to certain parts of the Bayesian workflow, the numbering does not align perfectly with workflow progression.

To aid you on this journey, we provide a list of 10 tips, summarized in Fig 1, to help you get started with Bayesian statistics. In Table 1 we have also compiled a glossary for definitions of technical terms. Our paper is not intended as a comprehensive introduction to Bayesian statistics. Instead, it provides guidance for applying Bayesian statistics and points to additional resources where you can learn the basics. This paper is not just for newcomers but also for those with some experience in Bayesian methods who may use it as a roadmap to design, conduct, and publish Bayesian analyses. We’ve drawn mainly on our experience teaching and working with ecologists, but we hope these tips will be relevant to a broader audience of biologists. For those seeking to deepen their understanding, we point to more comprehensive resources that offer an in-depth exploration of Bayesian statistics. The purpose of our paper is not to persuade you to abandon frequentist methods in favor of Bayesian methods. Instead, we advocate for a pragmatic dual approach where you master both methods as part of your analytical toolkit and choose the most appropriate tool for your problem.

**Table 1:** Glossary of terms used in Bayesian statistics.

Bayes factor	A statistical metric used to compare two competing models or hypotheses in a Bayesian framework. The Bayes factor is the ratio of the marginal likelihoods of the two models, which quantifies how much more (or less) the data support one model over the other. Marginal likelihoods are the probabilities of the observed data under a specific model, averaged over all possible values of the model parameters, weighted by their prior distributions. They capture both the goodness-of-fit and the complexity of the model. The Bayes factor is calculated as BF = *P*(data | Model 1)/*P*(data | Model 2). If BF > 1, the data provide more support for Model 1. If BF < 1, the data provide more support for Model 2. A BF close to 1 indicates little evidence favoring one model over the other.
Bayes’ rule	A derivation of the definition of the conditional probability, which in general can be written as *P*(*A* | *B*) = *P*(*B* | *A*)*P*(*B*)/*P*(*B*) and which can be used for non-Bayesian calculations when applied to observable quantities, e.g., in clinical testing. In Bayesian statistics, Bayes’ rule is used for inferences about unknown, unobservable quantities, and especially for parameters of a statistical model. Denoting parameters theta and a data set as *y*, Bayes’ rule can then be written as *P*(theta | *y*) = *P*(*y* | theta)*P*(theta)/*P*(*y*). Ignoring the constant in Bayes’ rule, we can also write this as *P*(theta | *y*) prop. *P*(*y* | theta)*P*(theta), i.e., the posterior is proportional to the product of the likelihood and the prior. Evaluation of *P*(*y*) often requires intractable integrals over all parameters and didn’t allow the application of Bayes’ rule for most practical applications for centuries. The discovery of simulation algorithms (MCMC; see below) circumvented this impasse and led to the great advance of Bayesian statistics observed during the last 30 years.
Bayesian model	A statistical model for a data set in which we combine a likelihood with priors that we chose for each parameter. Note that a Bayesian model is not fundamentally different from its corresponding non-Bayesian analog except for the priors. Thus a linear regression model (in terms of its likelihood) is the same whether we fit it using least-squares, maximum likelihood, or Bayesian posterior inference.
Bayesian statistics, also Bayesian inference or Bayesian posterior inference	The use of conditional probability, via Bayes’ rule, to update one state of knowledge using the information contained in some data set and embodied by the likelihood function and to arrive at a new state of knowledge, usually with reduced uncertainty about parameter and other estimated quantities.
Conditional probability	The probability of one RV given (‘conditioned on’) the known value of another RV, e.g., *P*(mass = 700 | sex = male), which gives the probability density of the mass of an animal of 700 g, given that it is a male. In general, written as *P*(*A* | *B*) for ‘probability of *A* given *B*’. This is defined as *P*(*A* and *B*)/*P*(*B*), i.e., as the joint probability of *A* and *B*, divided by the marginal probability of *B*.
Initial value	Numerical starting value for the MCMC that is defined for each parameter and should be different for each MCMC chain. Can be set manually as a fixed number or random draw from a specified distribution. Initial values not specified by the user may be filled in by the software before starting the MCMC, but the degree to which software does this (well) varies. “Bad” initial values, i.e., initial values that are not compatible with the model and/or the data generate initialization problem, which are among the most commonly encountered issues with Bayesian model fitting.
Joint density of a data set under a model	The joint density of obtaining the observed values of all data (and possibly random effects in the case of random-effects models) under a statistical model. Expressed in terms of the PDFs or PMFs of the model.
Joint probability	The probability of a combination of two (or more) RVs, or *P*(*A* and *B*).
Leave-one-out cross-validation (LOO-CV)	A technique for evaluating the predictive performance of a statistical model. In LOO-CV, the model is repeatedly trained on all the data except for one observation, which is then used to test the model’s prediction. This process is repeated for every observation in the dataset, and the results are combined to assess the model’s overall predictive accuracy. LOO-CV is particularly useful for assessing how well a model generalizes to new data. In Bayesian statistics, efficient approximations like Pareto-smoothed importance sampling (PSIS-LOO) are often used to speed up the process.
Likelihood function	Joint density function of all data under a model, when viewed as a function of the parameters. Represents the formal connection between data and parameters or loosely also the statistical model fitted to a data set.
Marginal probability	The probability of a random variable averaging over (or integrating over all possible values of) another random variable.
Markov chain Monte Carlo (MCMC) algorithm	A vast family of iterative algorithms that are typically used to fit Bayesian models. In essence, they function like random number generators (RNGs) for the posterior distributions that arise from Bayes’ rule when combining the likelihood of the data under a model and the priors chosen for the model’s parameters. These distributions can be approximated to an arbitrary degree of accuracy by drawing increasing samples of all parameters.
Maximum likelihood	A principle that says that the best possible ‘guess’ for a parameter is that value which maximizes the likelihood function when evaluated for the observed data set.
Outcome	A possible value of the result of a random experiment, e.g., the numbers 1–6 when tossing a die.
Posterior, also posterior distribution	A statement of how likely different values are for a parameter in a Bayesian model when we incorporate the information in our data set, i.e., *P*(theta | *y*). This is another proper probability distribution that integrates to 1 over the parameter space.
Posterior model probability	The probability that a particular model is the true model, given the observed data and the prior information. It is calculated using Bayes’ theorem, combining the model’s prior probability (belief about the model before seeing the data) and its marginal likelihood (how well the model explains the data). Posterior model probabilities provide a quantitative measure to compare models, with higher values indicating greater support for a model given the data and priors. They are especially useful in Bayesian model averaging and model selection.
Prior, also prior distribution	A statement of how likely different values are for a parameter in a Bayesian model before any information in a data set to be analyzed is incorporated, *P*(theta). This is a probability distribution, thus it integrates/sums to 1 over the entire parameter space.
Prior predictive distribution	This is *P*(*y*) in Bayes’ rule and is the probability distribution of the data when averaged over all possible values of the priors. When evaluated for a given data set, also called the ‘normalizing constant’ since it ensures that the integral of *P*(theta | *y*) becomes equal to 1. The value of the normalizing constant is obtained by integration over all parameters of the model, which in practice can hardly ever be done. MCMC algorithms circumvent this.
Probability density function (PDF)	Probability distribution for a continuous RV. It gives the probability density for any possible value *x* of the continuous RV, which corresponds to the area under the curve of a rectangle with basis (*x* − *d*, *x* + *d*) as d goes towards zero. Typical examples: normal (or Gaussian), exponential.
Probability distribution	Mathematical function that assigns a probability (for the PMF) or a probability density (for the PDF) to all possible values of a RV. Sums to 1 for a PMF and integrates to 1 for a PDF.
Probability function	Function that assigns a probability, or a value between 0 and 1, to any outcome of a random experiment. Values 0 and 1 denote respectively an impossible and a certain outcome.
Probability mass function (PMF)	Probability distribution for a discrete RV. It gives the probability of every possible value that the RV can take. Typical examples: Poisson, Binomial.
Random experiment	Taking a measurement or observation that has some stochasticity associated. E.g., tossing a coin, capturing an animal and measuring its mass.
Random variable (RV)	Usually defined as a real-valued function defined on the outcome of a random experiment. In practice, something unknown that we want to estimate or the probability of which we want to evaluate in a statistical analysis. Continuous random variables (RVs) include measurements of durations, lengths, weights, and (in Bayes) the values of most parameters, while discrete RVs include counts or labels such as dead/alive, red/blond/brown, and (in Bayes) the values of discrete latent parameters such as abundance or presence/absence.
Reversible Jump MCMC (RJMCMC)	An extension of the MCMC method used for Bayesian model selection and transdimensional sampling. Unlike standard MCMC, which operates within a fixed parameter space, Reversible Jump MCMC allows the exploration of models with different numbers of parameters or structures (varying model complexity). It achieves this by “jumping” between parameter spaces of different dimensions. For example, in a regression with explanatory covariates *A* and *B*, the algorithm “jumps” between models with different combinations of *A* and *B* (e.g., models including only *A* or *B*, or the two covariates). By doing so, it estimates both the parameters for each model and the posterior probability of each model being the best fit for the data.
Statistical model	A set of PDFs/PMFs for all observed random variables (i.e., data) or unobserved random variables (i.e., random effects, latent variables).
Widely applicable information criterion (WAIC)	A statistical tool used to compare different models based on their ability to predict new data. WAIC balances the model’s goodness-of-fit (how well it explains the data) and its complexity (to avoid overfitting). It is calculated using the likelihood of the observed data and includes a penalty for model complexity. Unlike simpler criteria like AIC, WAIC is fully Bayesian, accounting for uncertainty in the model parameters. A lower WAIC value indicates a better model for predictive accuracy.

### 1. Get a basic understanding of the ideas of Bayes

Bayesian statistics infers unknowns, such as model parameters, using conditional probability [3–5]. Probability in classical statistics describes the variability of observable data, treated as random variables with distributions. In Bayesian statistics, however, probability quantifies our knowledge about anything unobservable, including parameter values. For example, one might say, “I am 99% certain it will rain.” Conditional probability via Bayes’ rule allows for us to update prior knowledge (from independent data or assumptions) to a new state of knowledge represented by a posterior distribution.

**Fig 2:**
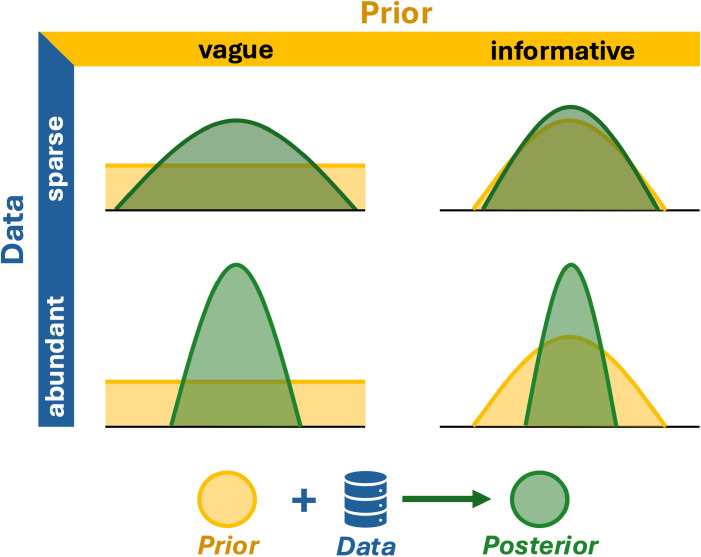
Schematic representation of prior (yellow) and posterior (green) distributions given data (blue). The prior distribution represents our expectation of what values the parameter can take. Vague priors are relatively flat across the possible parameter space (i.e., they contain little information), meaning that the posterior distribution is based primarily on information contained in our data. Informative priors can be used to include auxiliary knowledge (e.g., from the literature or expert judgements) to be evaluated alongside data when determining the posterior distribution and can be useful particularly in situations with sparse data.

The likelihood function is defined by the statistical model; in a way, the likelihood function *is* the model—it is the link between the data and the parameters being estimated [6]. It underpins Bayesian and frequentist methods alike, but while frequentists rely solely on likelihood for estimation, Bayesians also incorporate prior distributions (Tip #2), allowing external information to influence the estimates. A Bayesian model thus combines a likelihood with priors, resulting in the posterior distribution which reflects our updated knowledge of a parameter as a probability distribution (Fig 2). Point estimates can be obtained using summary metrics like the mean, median, or mode, and uncertainty via standard deviation or percentiles.

Most Bayesian models require approximations to evaluate Bayes’ rule, commonly through simulation techniques such as Markov chain Monte Carlo (MCMC). MCMC generates a sequence of random values for each parameter that starts from a specified initial value and ideally converges on a stationary distribution that is the desired posterior distribution. The magic of MCMC simplifies the otherwise complex or even intractable integrals involved in Bayes’ rule by sampling values proportional to their posterior distribution.

### 2. Think of your priors

The prior distribution is a fundamental part of Bayes rule and any Bayesian model. The prior describes our expectations regarding the possible values for a parameter, and allows us to formally incorporate auxiliary information in the analysis (via an informative prior). Nonetheless, most analysts specify priors that express a lack of prior information (“vague” or “noninformative”)—or, as is often said, to “let the data speak for themselves” — even if auxiliary information is available from previous studies or expert opinion (Fig 2). There are some situations in which using informative priors is reasonable or even necessary [7,8]. For example, when a complex model has a known non-identifiable or weakly identifiable parameter (Tip #6), the use of an informative prior may make this parameter estimable. Another example is using parameter estimates from previously conducted, comparable studies to improve estimates (chapter 20 in [9]).

So, exactly *which* prior distribution do we choose for a parameter? For vague priors, you want a distribution that (i) is “locally uniform” (approximately flat) in the vicinity of the true value of the parameter and (ii) covers the full range of its permissible values. For example, for regression parameters (intercept, slopes), we could use a normal distribution with mean 0 and large variance, whereas for probabilities, a uniform distribution between 0 and 1 is a natural choice. There are nonetheless many alternatives for specifying either vague or informative priors for different parameters, and the “right” choice will depend on the model, data, and auxiliary information available [8,10]. The effect of prior choice on inferences can be checked with a prior sensitivity analysis—if there is concern whether a specific prior is sufficiently vague compared to some other choice, test them against each other by rerunning the analysis and see how (much) the results change.

### 3. Apply standard modeling principles

General statistical principles still apply in Bayesian statistics [11,12] (Appendix B in [13]), e.g., always clarify your modeling goals (whether to describe, understand, explain, or predict), show/visualize your data [14], check assumptions, plan your study design carefully [13], and report effect size. In this tip, we focus on two such principles.

Start simple, increase complexity little by little: Sometimes people get into Bayesian analysis because they have a complex modeling problem they cannot solve with a canned R, Python, or Julia package [15]. Upon learning how to write models in one of the languages compatible with Bayesian software, it is tempting to jump straight in and try to implement that complex model in the Bayesian framework of your choice.

In doing so, there is a good chance you will wait for half an eternity, only to be confronted with various arcane errors that are hard to make sense of or worse, the software might not generate errors, but the estimates, while appearing to be drawn from a stationary distribution yet, are in fact stuck in a corner of the posterior distribution. Avoid this by following one simple rule: always start from the simplest possible version of your model! That is, you should take a modular, stepwise approach to model fitting—this allows you to test whether “things are in order”: your data are formatted properly, there are no strange data issues such as data values out of range, or bad prior distributions that suggest inconsistent data.

This stepwise approach is especially important in Bayesian analysis because the performance and behavior of the MCMC algorithm is paramount. MCMC runtime scales with the size of your data set *and* model complexity, so starting with a simple model allows you to benchmark the run-time and to check that it is viable within the scope of your work plan (e.g., [16]). Moreover, as the model increases in complexity, it will be easier to identify potential data limitations or identifiability problems (Tip #6). After this first, simple model is running well and the results are sensible, you can incrementally build your model up to be more and more complex until arriving at your desired model (Tip #7).

Use simulated data sets: a complementary approach to get an even better grasp of your model is to work with simulated data sets. Data analysis and data simulation are almost the same thing, but use a model in different directions: analysis takes data and models and estimates parameters, while simulation assumes parameter values and a model and generates potential data sets.

The first goal of data simulation is to enforce an understanding of a model: if you are unable to simulate data under a model, you probably don’t fully understand it. Similarly, computer code for data simulation under a model is arguably a superb and underutilized method to explain a statistical model to non-statisticians.

Additional advantages of data simulation include [17,18]: (1) Truth is known, hence, you can validate your model or your code to fit it. (2) It may help you understand complicated statistical concepts. For instance, if you don’t understand what a standard error is, you can repeatedly simulate a data set, estimate some parameter and realize that the standard error is simply the standard deviation of these estimates over replicates. (3) You can evaluate bias and precision of your estimators. (4) Power analysis evaluates the probability with which you can reject a null hypothesis in a significance test, and data simulation is the most general manner of evaluating power. (5) Study design (e.g., what’s the minimum required sample size) is also best approached with simulated data. (6) To check the robustness of a model to assumption violations, we can simulate data under a more general model and then fit a simpler model that lacks crucial assumptions.

### 4. Check model performance

Working with Bayesian statistics is an iterative process consisting of multiple rounds of building, assessing, and revising models (Tip #3). Model assessment typically has several steps too, and most focus on either MCMC performance or model goodness-of-fit (GOF; Tip #5). Obtaining reliable inferences from a Bayesian model requires that we run multiple chains and assess their convergence and adequate mixing to provide us with valid posterior samples.

**Fig 3:**
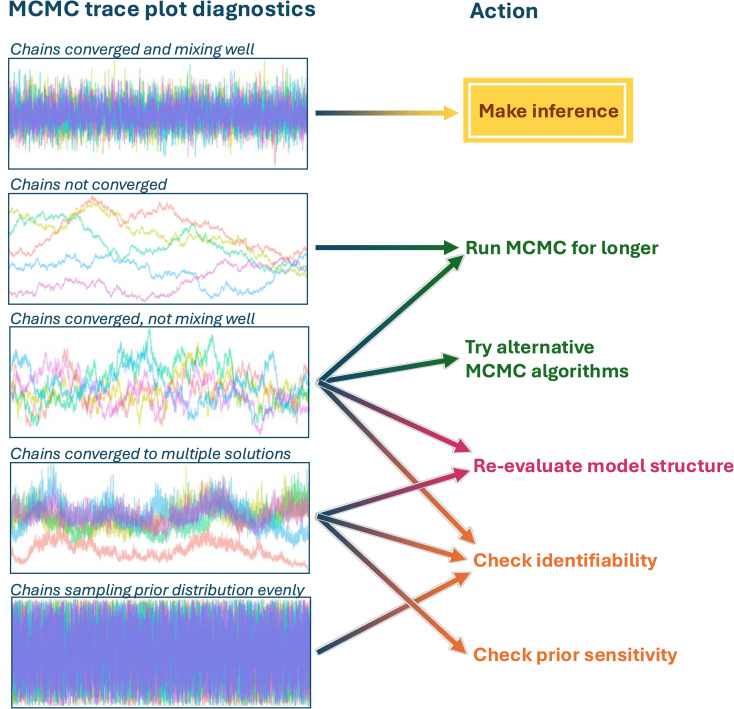
Overview of commonly observed patterns in MCMC trace plots and how to interpret them for diagnostics. The *x*-axis gives the iteration number and the *y*-axis gives the value sampled for a parameter. The colors of the writing link back to the color-coding of the steps in Bayesian modeling workflow depicted in Fig 1. Converged chains look like they are stable and fluctuating around a single average. Well-mixing chains have a high degree of overlap with each other. Suboptimal mixing, chains converging to different solutions, and chains that—at convergence—still sample the entire parameter space defined by the prior distribution often indicate problems with the model that warrant further investigation.

“Convergence” implies the Markov chains have stabilized and samples are being generated from the desired posterior distribution. This is required for valid inferences from the random numbers produced by an MCMC algorithm. “Mixing” refers to the degree to which different MCMC chains sample the same parameter space. Visualization is one of the best tools for Bayesian model assessments in general [14] and time-series, or trace plots, of the MCMC chains allow us to check convergence and mixing and subsequently adapt our model accordingly (Fig 3).

Non-convergence can often be addressed by running the algorithm for more iterations. Poor mixing can sometimes be improved by reparameterization (chapter 12 in [19]), or selecting different MCMC algorithms, but may also suggest underlying model issues that need addressing, e.g., non-identifiable parameters (Tip #6) or assumption violations. Quantitative metrics for checking MCMC convergence and chain mixing should complement visualizations, e.g., the potential scale reduction statistic R-hat [20], which measures divergence in the behavior of multiple chains.

### 5. Evaluate model goodness-of-fit

How well a model fits the data is crucial for the trust you can put into the parameter estimates it produces. GOF tests are well-established in frequentist statistics, and many can be applied to simple Bayesian models (e.g., residual analysis). However, Bayesian methods are often used for more complex models, for which we lack simple off-the-shelf GOF tests [21].

Posterior predictive checks, which simulate new datasets from the model’s posterior and compare them to the actual data, are commonly used. The more similar simulated and observed datasets are, the more likely the model fits well; this can be assessed visually and using a ‘Bayesian p-value’ [14,22]. Prior predictive checks, based on prior distributions alone (without using data from the analysis), assess adequacy using subject-matter knowledge [23] and may suggest models (especially priors) are inadequate when they predict impossible data (e.g., negative body weight, fewer animals alive than killed per year).

Last but not least: expert knowledge is essential for model checking. The first stage in model checking should be asking the question: “Do the estimates make biological sense?”. If the answer is no, the model needs revising.

### 6. Beware of model non-identifiability

The Bayesian approach and MCMC methods enable the construction of complex models, but may risk over-parameterization and non-identifiable parameters—those that are confounded and not independently estimable [24]. Models with non-identifiable parameters often show poor MCMC mixing and may converge slowly. However, detecting non-identifiability can be challenging, as parameters may appear estimable from available data even when they are not [24].

To diagnose non-identifiability, simulations are powerful (Tip #3). An inability to recover true parameter values from simulated data may suggest potential non-identifiability, as can a high correlation between MCMC samples for two parameters in pairwise scatter plots. Examination of the overlap between prior and posterior distributions is helpful because non-identifiable parameters often show substantial overlap and limited ‘Bayesian learning’ [25]. However, large overlap may also result from an informative prior aligning with the posterior, hence overlap alone cannot confirm non-identifiability. Frequentist methods should not be forgotten. For instance, profiling the likelihood [26] involves maximizing a likelihood with respect to all parameters except one, held constant at a range of values. A flat profile likelihood indicates non-identifiability for that parameter. Other methods using symbolic algebra can diagnose non-identifiability, but require advanced expertise and may quickly become impractical with many parameters [24].

If a model is not identifiable, you will likely have to simplify the model structure (Tip #3) until the issue is resolved. In addition, you should consider reparameterizing your model [26–28]. If the problem still persists, evaluate the possibilities for using informative priors to provide additional information for non-identifiable parameters [27,29–31] or collecting additional data as required for an identifiable model.

### 7. Compare models

In the hypothetico-deductive framework, models are compared to evaluate the relative strength of evidence in the data supporting alternative hypotheses. In Bayesian statistics, this is achieved by assessing models based on their probability of being true given the data, characterized by the posterior model probability.

An approach to derive these probabilities is through Bayes factors, but this approach can be computationally demanding, and particularly sensitive to priors [32], which has limited its use in practice [33]. An alternative is to compute posterior model probabilities using reversible jump MCMC, a relatively straightforward approach to implement when selecting among a set of explanatory covariates.

When selecting models, it is essential to consider the goals of your analysis [34]. Is the focus on understanding underlying mechanisms (inference) or on prediction? A model with the highest posterior probability might not always be the best for predictive purposes. For model selection based on predictive ability [35], you can evaluate predictions either in-sample (using the same data the model was fit to) or out-of-sample (using new data). The latter is generally considered the most reliable approach but requires splitting your data into subsets for fitting and prediction. As a shortcut, the predictive ability can be estimated in-sample using methods like the widely applicable information criterion [36] and leave-one-out cross-validation [37].

Alternatively, one might forego Bayesian model selection altogether. Instead, a single comprehensive model can be constructed and refined iteratively through fitting, testing, and critiquing steps [38,39]. This approach emphasizes exploring model variants to understand the system better, rather than choosing a single “best” model.

### 8. Know your tools

Just as you wouldn’t typically write optimization code from scratch to fit a model in the frequentist framework, we recommend using established software with reliable, well-tested, and optimized MCMC algorithms. We focus on free options here, and there are numerous choices for Bayesian statistics available in R, Python, and Julia [15].

Selecting software based solely on speed and efficiency is challenging [40], as it involves more than just measuring raw computation time. You also need to factor in your experience level and (un)familiarity with a modeling language.

Bayesian software generally falls into two categories: general-purpose model-fitting engines requiring coding such as WinBUGS/OpenBUGS [41], JAGS [42], Stan [43], or NIMBLE [44], and those with built-in models such as JASP [45], PyMC [46], or brms [47]. Coding offers theoretically unlimited flexibility for custom models, but non-coding options handle many tasks with minimal effort. Prioritize getting the code to work before optimizing for performance. Custom R packages such as brms offer ease of use, often adopting standard R conventions for model specification. On the other hand, MCMC engines such as JAGS, Stan, and NIMBLE offer flexibility to describe novel models of extraordinary complexity, and enhance understanding of the different steps of a Bayesian analysis.

For a beginner, it may be wise to pick one software and stick with it, as familiarity helps with debugging, knowledge of useful tricks, and implementing advanced techniques. However, as you gain confidence, consider diversifying your toolkit to tackle tasks that may be challenging with your default software.

### 9. Embrace reproducibility

Awareness of reproducibility’s importance is growing in the biological sciences [48,49], with publishers and funders emphasizing the publication of well-documented data and code [50]. Modern research workflows begin with study design and data collection, and end with presenting results. Ensuring reproducibility increases the quality and credibility of your work, enhances efficiency in debugging and re-running analyses, and enables broader use of your model and indeed your entire workflow (Tip #10). Given the randomness inherent to MCMC and the many steps and decisions in Bayesian workflow (Fig 1), considering reproducibility from the start—not just as an afterthought—could become a superpower in your work with Bayesian models.

Four pillars support reproducibility: (1) Control of randomness: Specifying seeds for random number generators when simulating data, setting initial values, and running MCMC ensures consistent results. This enhances reproducibility and simplifies debugging, especially with a complete set of initial values. (2) Clean coding [51]: Adopting best practices for writing code [52] helps others and yourself understand and re-run analyses later. Clean coding is also the first step in automating your workflow and facilitates running code on high-performance computing setups. (3) Good documentation means thoroughly annotating your code and documenting workflow steps, which benefits others and increases potential for broader impact. (4) Version control using git repositories (GitHub/GitLab) facilitates code development and management and promotes collaboration [53].

### 10. Learn and get help from others

Learning Bayesian statistics can be daunting. Fortunately, you are not alone on this journey. We recommend you engage with the Bayesian community within your field of research by leveraging available resources, such as online forums, workshops, and textbooks.

A good starting point is to identify an introductory textbook relevant to your field of biology that covers Bayesian statistics. To build confidence in your skills, consider conducting side-by-side analyses using both frequentist and Bayesian approaches [9,54–56]. This comparative practice can help you appreciate that numerical results often align. Begin with a simple project of your own or replicate a past analysis from your work or published studies.

If you have questions, don’t hesitate to seek help from forums such as Cross Validated or Stack Overflow or more specialized, subject-matter lists. For software-specific inquiries, consider reaching out through mailing lists or forums, and best include a small, reproducible example to illustrate your problem. The feedback you receive often benefits other beginners as well.

Attending workshops can be an excellent way to learn under the guidance of experienced teachers. These events also offer excellent networking opportunities. As you become more advanced, workshops provide avenues to master new methods and further enhance your skills.

Remember, modern science is a collaborative effort, and Bayesian statistics is no exception. If a task feels beyond your current expertise, seek collaboration to learn from others and achieve your research goals.

## Conclusion

Bayesian statistics has grown rapidly in biology, although it remains a technically challenging subject that many researchers had insufficient exposure to during their graduate studies. Learning a new skill can be demanding and time-consuming. While the material we discussed is not new and has been explored by others [2,57,58], our tips aim to offer short and practical guidance to help you get started. These insights are designed to support your journey through Bayesian statistics in biological research, regardless of your specific field. We believe that the effort in learning Bayesian statistics is highly warranted for an early-career scientist and over time will pay off many times over.
